# Measurements of dichroic bow-tie antenna arrays with integrated cold-electron bolometers using YBCO oscillators

**DOI:** 10.3762/bjnano.15.3

**Published:** 2024-01-04

**Authors:** Leonid S Revin, Dmitry A Pimanov, Alexander V Chiginev, Anton V Blagodatkin, Viktor O Zbrozhek, Andrey V Samartsev, Anastasia N Orlova, Dmitry V Masterov, Alexey E Parafin, Victoria Yu Safonova, Anna V Gordeeva, Andrey L Pankratov, Leonid S Kuzmin, Anatolie S Sidorenko, Silvia Masi, Paolo de Bernardis

**Affiliations:** 1 Nizhny Novgorod State Technical University n.a. R.E. Alekseev, Minin Street, 24, Nizhny Novgorod, 603155, Russia; 2 Institute for Physics of Microstructures of the Russian Academy of Sciences, Akademicheskaya Street 7, Nizhny Novgorod, 603950, Russia; 3 Department of Microtechnology and Nanoscience - MC2, Chalmers University of Technology, 41296 Gothenburg, Sweden; 4 D. Ghitu Institute of Electronic Engineering and Nanotechnologies of Technical University of Moldova, MD-2028 Chisinau, Republic of Moldova; 5 Dipartimento di Fisica, Università La Sapienza, I-00185 Roma, Italy

**Keywords:** cosmic microwave background, B mode, cold-electron bolometer, dichroic antenna array, dipole bow-tie antenna, Josephson junction, LSPE

## Abstract

We consider properties of dichroic antenna arrays on a silicon substrate with integrated cold-electron bolometers to detect radiation at frequencies of 210 and 240 GHz. This frequency range is widely used in cosmic microwave background experiments in space, balloon, and ground-based missions such as BICEP Array, LSPE, LiteBIRD, QUBIC, Simons Observatory, and AliCPT. As a direct radiation detector, we use cold-electron bolometers, which have high sensitivity and a wide operating frequency range, as well as immunity to spurious cosmic rays. Their other advantages are the compact size of the order of a few micrometers and the effect of direct electron cooling, which can improve sensitivity in typical closed-loop cycle ^3^He cryostats for space applications. We study a novel concept of cold-electron bolometers with two SIN tunnel junctions and one SN contact. The amplitude–frequency characteristics measured with YBCO Josephson Junction oscillators show narrow peaks at 205 GHz for the 210 GHz array and at 225 GHz for the 240 GHz array; the separation of these two frequency bands is clearly visible. The noise equivalent power level at an operating point in the current bias mode is 5 × 10^−16^ W/√Hz.

## Introduction

The cosmic microwave background (CMB) radiation contains a lot of information about origin and evolution of our universe. The temperature and polarization patterns of the CMB give us an accurate picture of the age of the universe and the evolution after the Big Bang [1]. It is currently assumed that the polarization of the CMB radiation can be decomposed into an E mode and a B mode. The more interesting part of the CMB radiation polarization is the B mode. It presumably originates from gravitational waves from the cosmic inflation, while the E mode originates from acoustic waves from the recombination. Currently, the following CMB experiments are in operation or in preparation: Background Imaging of Cosmic Extragalactic Polarization (BICEP) Array [2], Large Scale Polarization Explorer (LSPE) [3], Lite (Light) satellite for the study of B mode polarization and Inflation from cosmic background Radiation Detection (LiteBIRD) [4], Q & U Bolometric Interferometer for Cosmology (QUBIC) [5], Simons Observatory [6], and Ali CMB Polarization Telescope (AliCPT) [7].

The BICEP Array is a successor to the Keck Array from the BICEP series. It is a radio telescope installed at the South Pole research station. This telescope is aimed at CMB radiation polarization measurements, in particular, the measurements of the B mode. The Keck Array has been operating since 2012, and work on the BICEP Array was started in 2018. The BICEP Array is planned to observe the polarized microwave sky at 30/40, 95, 150, and 220/270 GHz. It is stated that the 220/270 GHz channel will have more than 20000 detectors. It will use radio frequency (RF) multiplexing with microwave superconducting quantum interference device (SQUID) readout if transition-edge sensors (TESs) detectors are installed. Otherwise, on-wafer RF multiplexing may be used with thermal kinetic inductance detectors [2].

The LSPE mission [3] is a project of the Italian Space Agency aimed at studying the polarization of the B mode of the CMB radiation. The antennas for frequencies of 210 and 240 GHz that we are studying may be used for the Short Wavelength Instrument for the Polarization Explorer (SWIPE) instrument of this project. SWIPE is a radio telescope mounted on a balloon. It has three frequency channels, one main channel at 145 GHz with 30% bandwidth, and two auxiliary channels at 210 GHz with 20% bandwidth and at 240 GHz with 10% bandwidth [3]. It is planned that multimodal TESs with spider-web antennas will be used as detecting elements in the current implementation of this project [8]. These detectors are to be installed into waveguide horns, which is a standard way to form the needed radiation pattern for the antenna. The main channel has 162 detectors, and each of the auxiliary channels has 82 detectors. It is intended to use SQUID readout [9–10] with frequency multiplexing for this mission [8]. The estimated photon noise equivalent power (NEP) for the auxiliary channels is about 2 × 10^−16^ W/√Hz.

The LiteBIRD is a satellite mission that investigates the B mode polarization of the CMB radiation to test the hypothesis of an expanding universe. This mission is carried out by the Japanese Institute of Cosmonautics and Astronautics with the support of the Japanese Aerospace Exploration Agency. The LiteBIRD payload consists of three telescopes for low, mid, and high frequencies. Mid and high frequencies are treated as a whole. The focal planes of the telescopes are planned to be filled with TES bolometers. The low- and mid-frequency arrays consist of dual- and triple-frequency detectors combined with sinusoidal antennas and silicon lenses. The high-frequency array consists of single- and dual-frequency detectors with orthomodal transducers and silicon horns. This array is aimed at 195, 235, 280, 337, and 402 GHz, and the total number of detectors is 1350. The channels at 195 and 235 GHz each have 254 detectors combined into 127 pixels, and the bandwidth is only 0.3 GHz for each channel. The estimated photon NEP for these channels is about 13 × 10^−18^ W/√Hz [4].

QUBIC [5] is an instrument for measuring the B mode polarization pattern of the CMB. It is a ground-based experiment planned to be placed in Argentina at Alto Chorrillos in the Santa province. This polarimeter is equipped with bolometric interferometers featuring self-calibration ability for systematic controlling of effects. This feature provides great spectral imaging capabilities to QUBIC. QUBIC is planned to observe the sky at 150 and 220 GHz with 25% bandwidth for both frequency channels. There will be 992 TES detectors for each channel on the focal plane. The estimated NEP for this setup is 4.7 × 10^−17^ W/√Hz. But because of aliasing in the SQUID readout system and microphonic noise, the NEP in first QUBIC tests was limited to 2 × 10^−16^ W/√Hz.

The Simons Observatory [6] is a four-telescope ground-based system, including one large-aperture telescope and three small-aperture telescopes. It will be built in Chile in the Atacama desert to study the evolution of the late universe using the Sunyaev–Zeldovich effects. Three frequency bands are used in the Simons Observatory: 30/40, 90/150, and 220/280 GHz. They will be separated by universal focal-plane modules consisting of 432 pixels. It is planned to use TESs as detectors coupled to the optics and microwave-multiplexing SQUID readout. The 220/280 GHz band will have 1728 active detectors and 36 so-called “dark” detectors, used for calibration, per array.

The AliCPT [7] is a ground-based experimental setup aimed at CMB measurements in Tibet, China, at an altitude of more than 5000 m. Tibet is known as one of the best places for CMB observations on the Northern Hemisphere. AliCPT is planned to observe the sky at frequencies of 95 and 150 GHz. AliCPT will use TESs as detectors; there are 1712 of them on a single focal plane, and the total number of detectors will be more than 27000 in the final setup. It is stated that AliCPT will have one of the most sensitive detectors in the world. The total expected NEP of this system is about 6.77 × 10^−17^ W/√Hz, and the required NEP for this experiment should be below 7.5 × 10^−17^ W/√Hz.

The concept of arrays of dipole antennas for frequencies of 210 and 240 GHz with cold-electron bolometers, described in the present paper, is proposed to be suitable for such applications. Two arrays placed on a single silicon chip with 7 mm × 7 mm size can independently detect radiation at two frequencies. Here, in the design of dichroic receiving systems, one should overcome the limitation of a silicon substrate, whose thickness affects the efficiency of detection as a refractive medium. In the case of close frequencies, however, one can find a compromise of matching the average frequency of both arrays with reasonable detection efficiency. As a direct radiation detector, we consider using the cold-electron bolometer (CEB) concept [11–12]. These bolometers have high sensitivity with background-limited operation [13–15], a broad operating frequency range, as well as immunity to spurious cosmic rays [16]. Since CEB sizes are of the order of a few micrometers, detector can be placed inside the antenna slot without additional microwave feed lines, forming a multi-absorber array [15,17–18]. Because of this feature, one can adjust the total resistance by forming series or parallel arrays of CEBs to match for either junction field-effect transistor (JFET) or SQUID readout.

The principal advantage of these CEB-based detectors over TESs [19] is the effect of direct electron cooling, when electrons with high energy are removed from a nanoabsorber, leaving only the quasiparticles with low energy and, accordingly, low electron temperature [20–23]. This advantage is important for space, balloon, and ground-based applications. While dilution refrigerators are able to cool down below 50 mK, they cannot operate in extreme or even slightly unstable conditions; even the small variation of a tilt angle can lead to the cryostat malfunction. Typical closed-cycle sorption ^3^He cryostats, suitable for space and balloon-borne experiments, are limited by their working temperature to about 300 mK. For example, the LSPE-SWIPE custom-designed ^3^He cryostat cools the two focal planes down to 0.3 K. The ground-based BICEP Array cryostat with a mixed ^4^He/^3^He/^4^He sorption fridge, working at arbitrary elevation angle, is able to achieve a temperature of 250 mK [2]. The AliCPT cooling system is similar to the one of the BICEP array with the same lowest temperature of 250 mK [7]. So, electron cooling is a pathway to improve sensitivity with efficient reduction of electron temperature down to 65 mK from a base temperature of 300 mK [22].

Here we present improved simulation results in comparison with [24] and the first results of fabrication and measurements, using YBa_2_Cu_3_O_7_ (YBCO) Josephson junction (JJ) oscillators, of a dichroic multiabsorber receiving system as a prototype for the 210/240 GHz auxiliary channels of the LSPE mission.

## Results and Discussion

### Numerical modeling of the antenna matrix with CEBs

**Figure 1:**
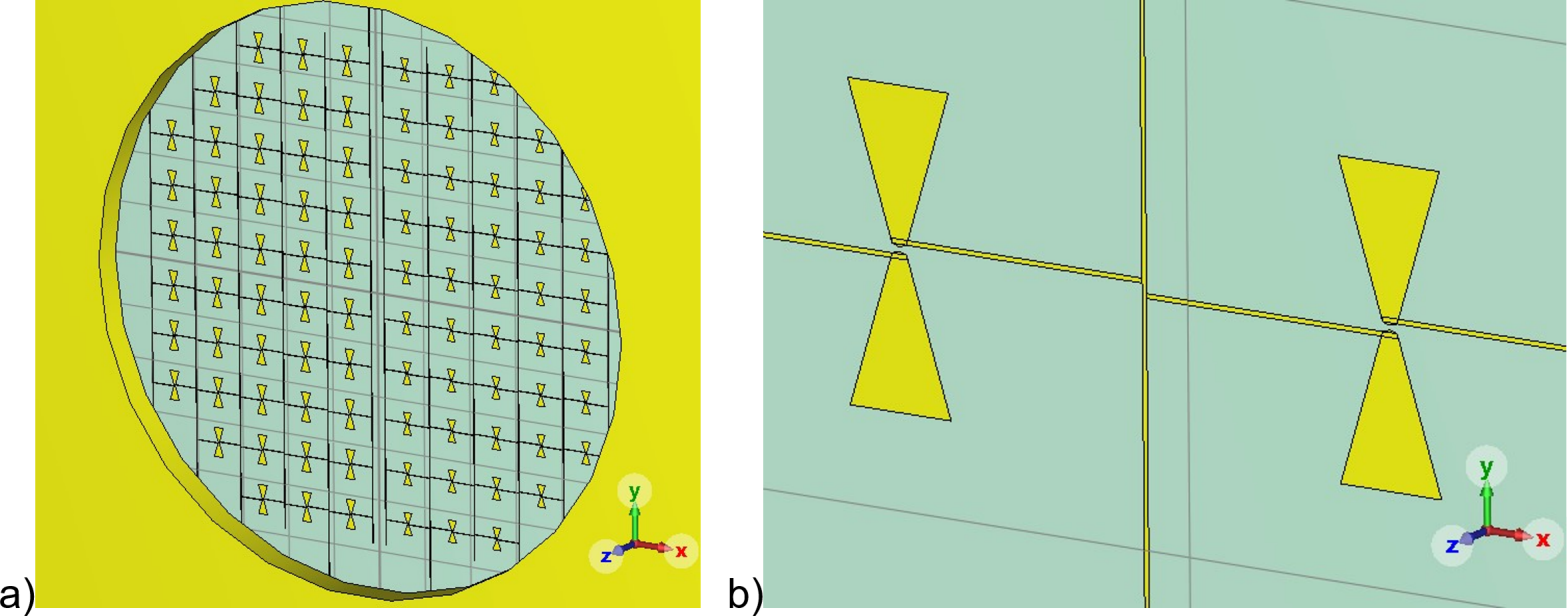
(a) A view of the matrix of dipole antennas with integrated CEBs. (b) Enlarged view of the receiving element of the antenna matrix consisting of 210 GHz bow-tie antennas with a CEB inside a slot.

As a receiving system prototype for LSPE, it is proposed to use a matrix of dipole antennas with integrated CEBs as sensitive elements [24] (Figure 1a). The basic element of the receiving matrix is a dipole bow-tie antenna, in the gap of which the CEB is embedded (Figure 1b). This matrix is located on a silicon substrate, which is 260 µm thick. Since the operation of the CEB in the matrix is assumed to be in the voltage bias mode, the elements of the receiving matrix are connected to each other in parallel.

The matrix of receiving elements will be located under the opening of the back-to-back horn, similar to the one used for the LSPE-SWIPE project, with a diameter of 4 mm. Accordingly, the receiving elements of the 210 and 240 GHz frequency channels occupy areas within the left and right semicircles, respectively (Figure 1a). In each semicircle there are 44 CEBs with bow-tie dipole antennas. The receiving elements are located on the side of the substrate that is turned to the incident radiation.

Numerical simulations of the frequency response of the two frequency channels of the receiving matrix were carried out. During the simulations, the antenna array was irradiated with the fundamental mode H_11_ of a round waveguide port with a diameter equal to the diameter of the opening of a back-to-back horn directed towards the receiving array. The fundamental mode of the circular waveguide was oriented so that the field E was directed parallel to the receiving dipoles. To exclude the effect of mode conversion upon partial reflection of the incident wave from the receiving matrix, and of subsequent reflection of higher modes from the waveguide port, this port was chosen as multimode with the number of modes equal to 35. As the equivalent circuit of the CEB in the calculation, a series RC circuit was used, the resistance of which was equal to the resistance of the CEB absorber, and the capacitance was equal to half the capacitance of a single SIN tunnel junction. The frequency response of the receiving matrix channel was calculated by summing up the power absorbed in each active resistance of the receiving element.

In the course of the work, the frequency response of the dipole antenna matrix with integrated CEBs was optimized by means of numerical simulation. The aim of the optimization was to bring the absorption bandwidth in line with the current requirements for the LSPE receiving system. According to these requirements, the width of the absorption line should be 20% of the operating frequency for the 210 GHz channel and 10% of the operating frequency for the 240 GHz channel.

**Figure 2:**
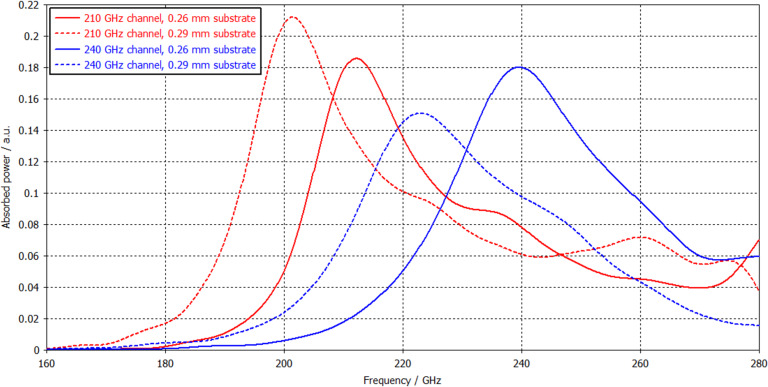
Frequency response of the two-frequency receiving system based on the matrix of bow-tie antennas with CEBs. Solid curves show the optimal response calculated at a substrate thickness of 0.26 mm, obtained by parameters optimization. Dashed curves calculated using a substrate thickness of 0.29 mm seem to be closer to the experimental results, see Measurement results section below.

In the process of optimization, the geometrical dimensions of the matrix dipoles, as well as the absorber resistance and the capacitance of the SIN tunnel junctions, were tuned to meet the requirements better. The best results have been achieved at the following values of the cell parameters: resistance = 15 Ω, capacitance = 25 fF, dipole length = 200 µm, and dipole width at the ends = 70 µm for the 210 GHz channel; resistance = 20 Ω, capacitance = 25 fF, dipole length = 150 µm, and dipole width at the ends = 61.25 µm for the 240 GHz channel. The period of the antenna array is 340 µm in both directions for 210 and 240 GHz channels. The frequency response of the two-frequency receiving matrix with a Si substrate thickness of 260 µm is shown in Figure 2 (solid curves). To compare the calculated response with the experimental results, we also added curves for a Si substrate thickness of 290 µm (dashed curves).

As a result of frequency response optimizations, the following characteristics were obtained: The bandwidth for the 210 GHz channel at half power level is 25.46 GHz, and the maximum absorption occurs at a frequency of 212.1 GHz; the bandwidth for the 240 GHz channel is 34.8 GHz, and the maximum absorption occurs at a frequency of 239.6 GHz.

### Fabrication of samples of receiving systems with CEBs

The samples and the sample blanks with electronic lithography, ready for electron beam evaporation, were fabricated at the Chalmers University of Technology (Sweden). The samples were deposited both at Chalmers University and, using the preforms, at our laboratory at Nizhny Novgorod State Technical University (NNSTU). The sample quality was analyzed using the diagnostic equipment of the Collective Use Center of the Institute for Physics of Microstructures of the Russian Academy of Sciences (IPM RAS), with subsequent measurements of the samples in the sorption ^3^He refrigerator of our laboratory. The 210/240 GHz receiving system is fabricated using a two-layer technology (two lithography steps). During the first photolithography step, a layer of contact pads, DC lines, and antennas is made. The second electronic lithography step is used for the exposition of the bolometric layer.

During photolithography, the first exposure was carried out with two photoresists. This is because the DC linewidth was 3 µm, and the use of a single photoresist would have resulted in jagged edges or a raised edge of the plating after lift-off, which in turn would have prevented thin electron beam resist from being deposited in the next operation. Therefore, LOR3A and S1805 photoresists were used. After the development, a layer of Ti/Au/Pd with thicknesses of 10/100/20 nm was deposited with an electron-beam evaporation machine.

**Figure 3:**
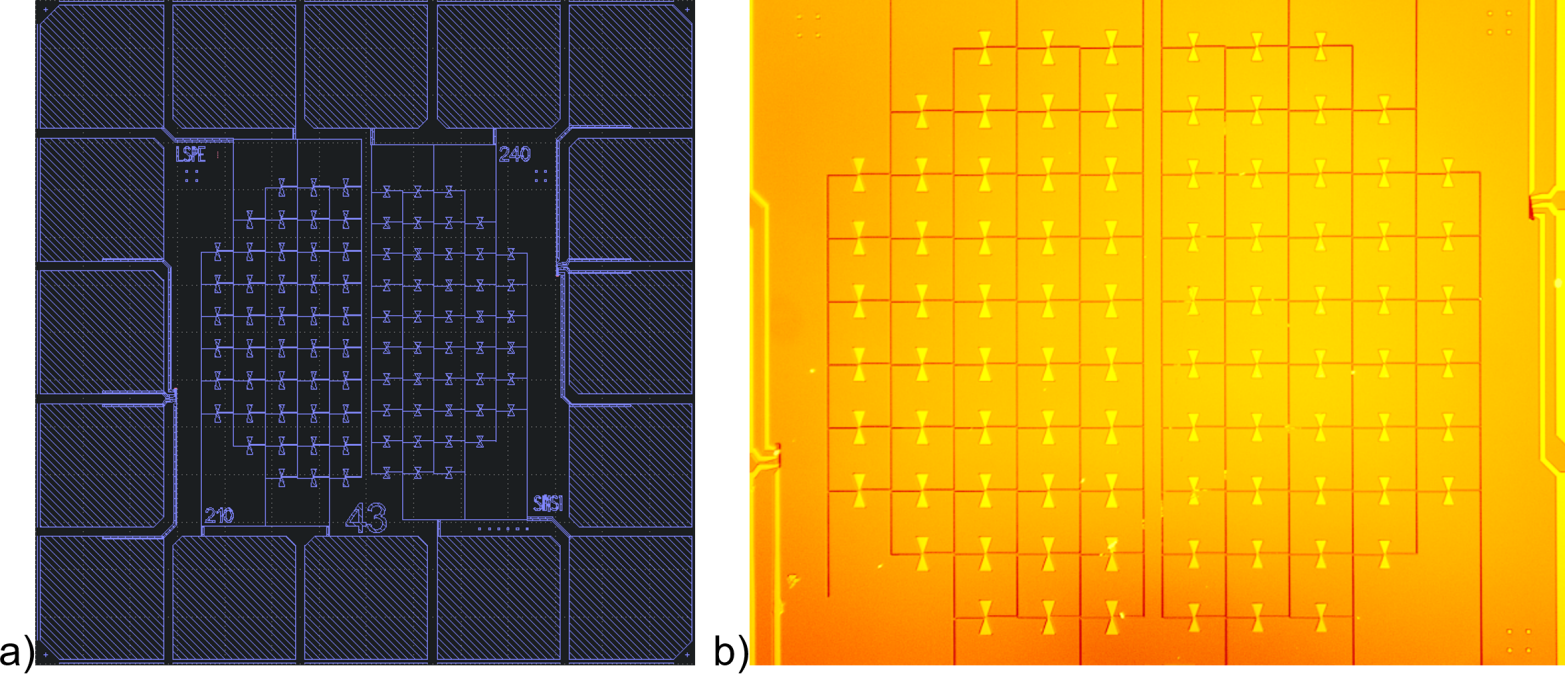
(a) Design of the sample of LSPE VB 210/240 from the SINS1 series. (b) Optical photo of the sample of LSPE VB 210/240 from the SINS1 series.

After lift-off, a double layer of MMA and ARP electron resist was deposited, which is necessary to form an aluminum oxide dielectric layer without breaking the vacuum. Then the electron lithography was made, but the resist was not developed on the samples to preserve the design imprint from getting damaged. In our laboratory of NNSTU, the samples were developed, and a bolometric layer, consisting of two SIN tunnel junctions and one SN contact, was fabricated using self-aligned shadow evaporation. The sample design (210/240 GHz dual-frequency receiving system prototype for the LSPE mission) is shown in Figure 3a, and a photograph from an optical microscope is shown in Figure 3b.

The shadow evaporation of the bolometric layer consists of the following stages: The first stage is the vertical deposition of a normal metal layer, consisting of 1.2 nm of Fe and 30 nm of Al on top; then, 60 nm of aluminum are deposited at an angle of 45°. Thus, we form a superconductor–normal metal (SN) Andreev contact. Subsequently, oxidation is carried out at a relatively high pressure (1–2 Torr) in the working chamber of the sputtering unit. The last stage is the deposition of about 70 nm of Al at an angle of −45° to form a SIN tunnel junction. Thus, as a normal layer we use the hybrid superconducting/ferromagnetic structure, which allows for decreasing the absorber volume and also for suppressing the Andreev heating current [22] to improve detector sensitivity.

**Figure 4:**
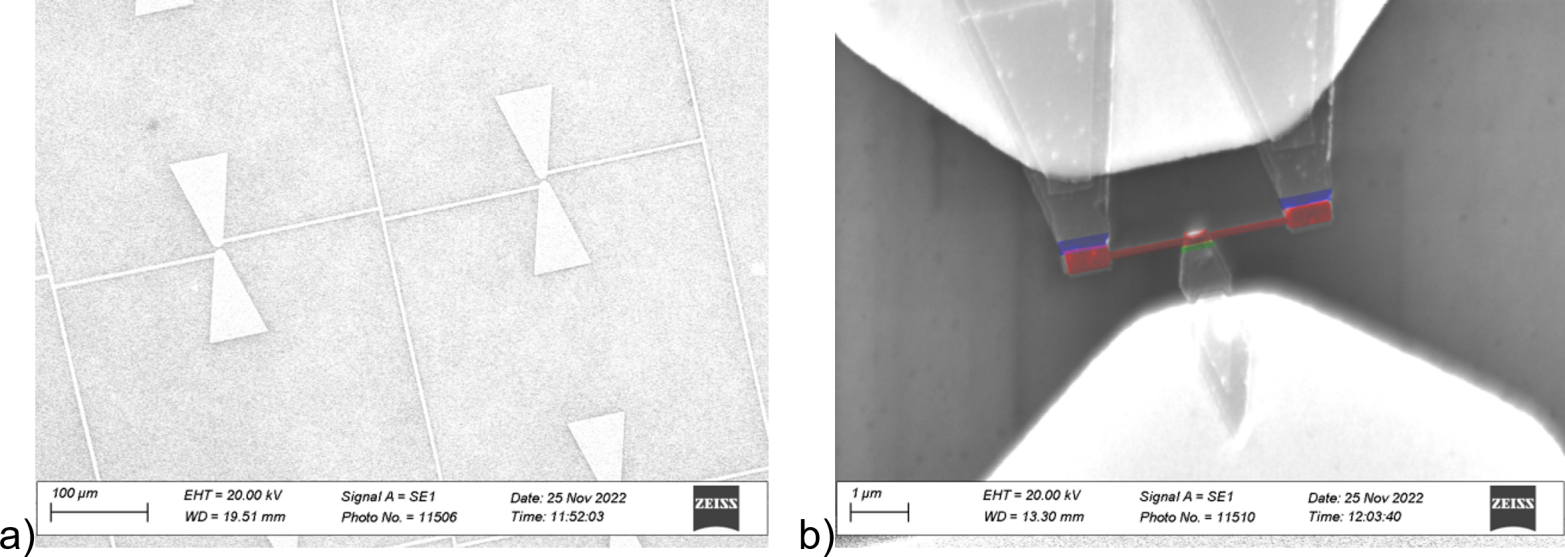
(a) SEM image of the LSPE sample; bow-tie antennas are visible. (b) SEM image where the required elements are painted with pseudocolors; red: normal metal absorber, green: SN contact, and blue: SIN tunnel junctions.

Scanning electron microscopy (SEM) images of the sample LSPE VB 210/240 SINS1 No. 43 deposited at NNSTU were obtained using an electron microscope at the Collective Use Center of the IPM RAS (Figure 4a,b). The dimensions of the junctions can be precisely measured using SEM and compared with the original layout.

The specific feature of this bolometer is the simultaneous presence of two SIN tunnel junctions and one SN contact (Figure 4b). This concept was invented [25] in order to reduce the resistance of the bolometer since the system was designed for SQUID readout, which is sensitive to the system resistance; also, the total noise is decreased. The SN contact should, in turn, accelerate the tunneling of hot electrons from the absorber, serving as an open gate.

In the course of measurements of the obtained samples, however, it turned out that the resistance of the obtained samples was higher than we had expected. Mainly because of the high resistance of the DC lines as well as the imperfection of the process of creating a barrier. Therefore, two more designs were created with an expansion of the DC lines in order to reduce the resistance. Also, adjustments were made to the oxidation process. In the future, it is planned to carry out a set of works to improve the barrier properties of aluminum oxide. The quality of the barrier largely depends on the roughness of aluminum. It can be critically large during electron beam deposition, which in turn affects the thickness of the barrier and the leakage resistance. We now test various ways to reduce the aluminum roughness. Also, the roughness strongly depends on the deposition rate. The oxidation process affects the barrier properties as well; perhaps with dynamic oxidation [26] (with constant pumping) one can try to achieve better results.

### Measurement results of samples of receiving systems with CEBs

**Figure 5:**
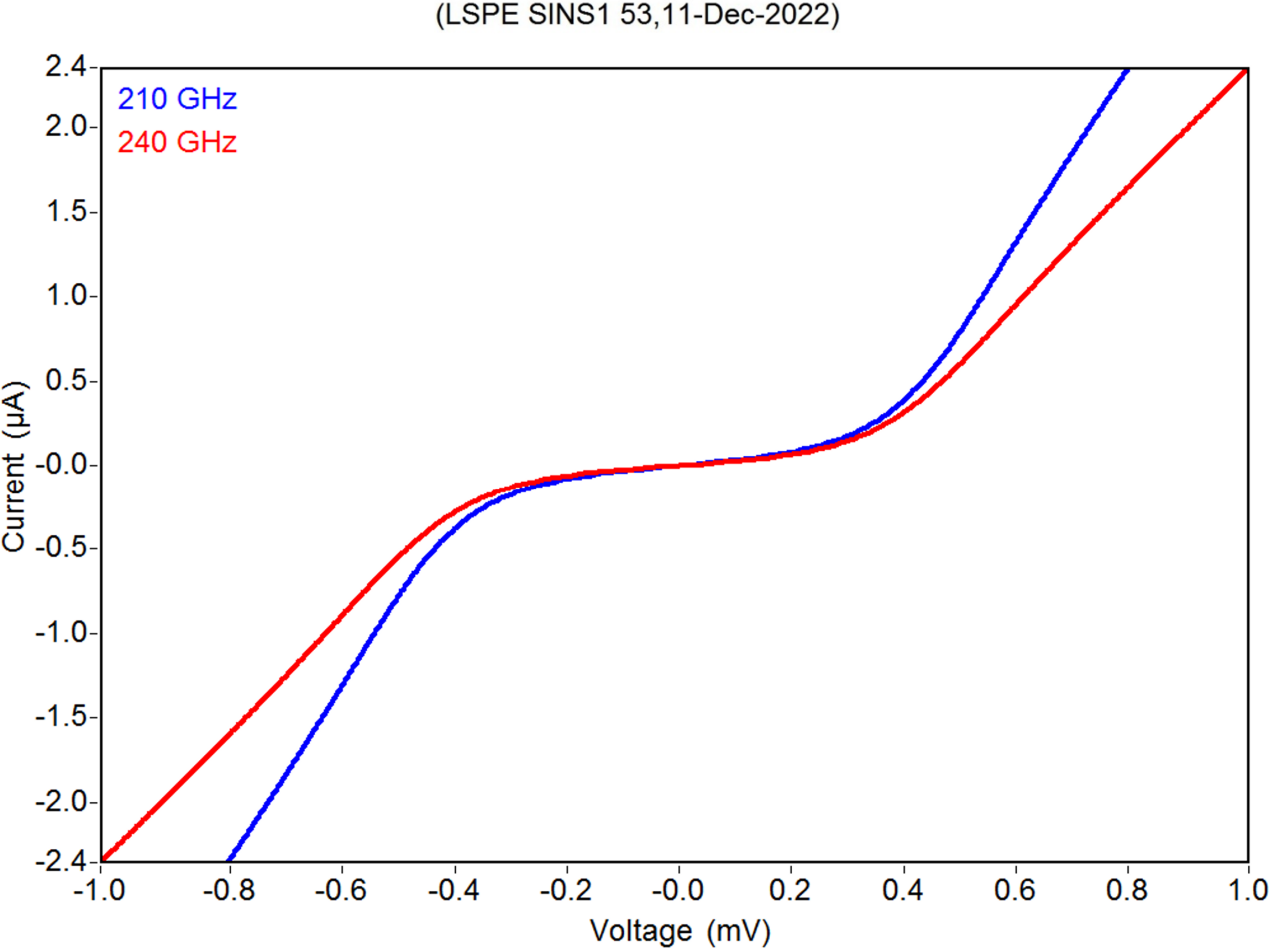
*I*–*V* characteristics of two receiving structures of a sample receiving system from the LSPE VB 210/240 SINS1 series.

**Figure 6:**
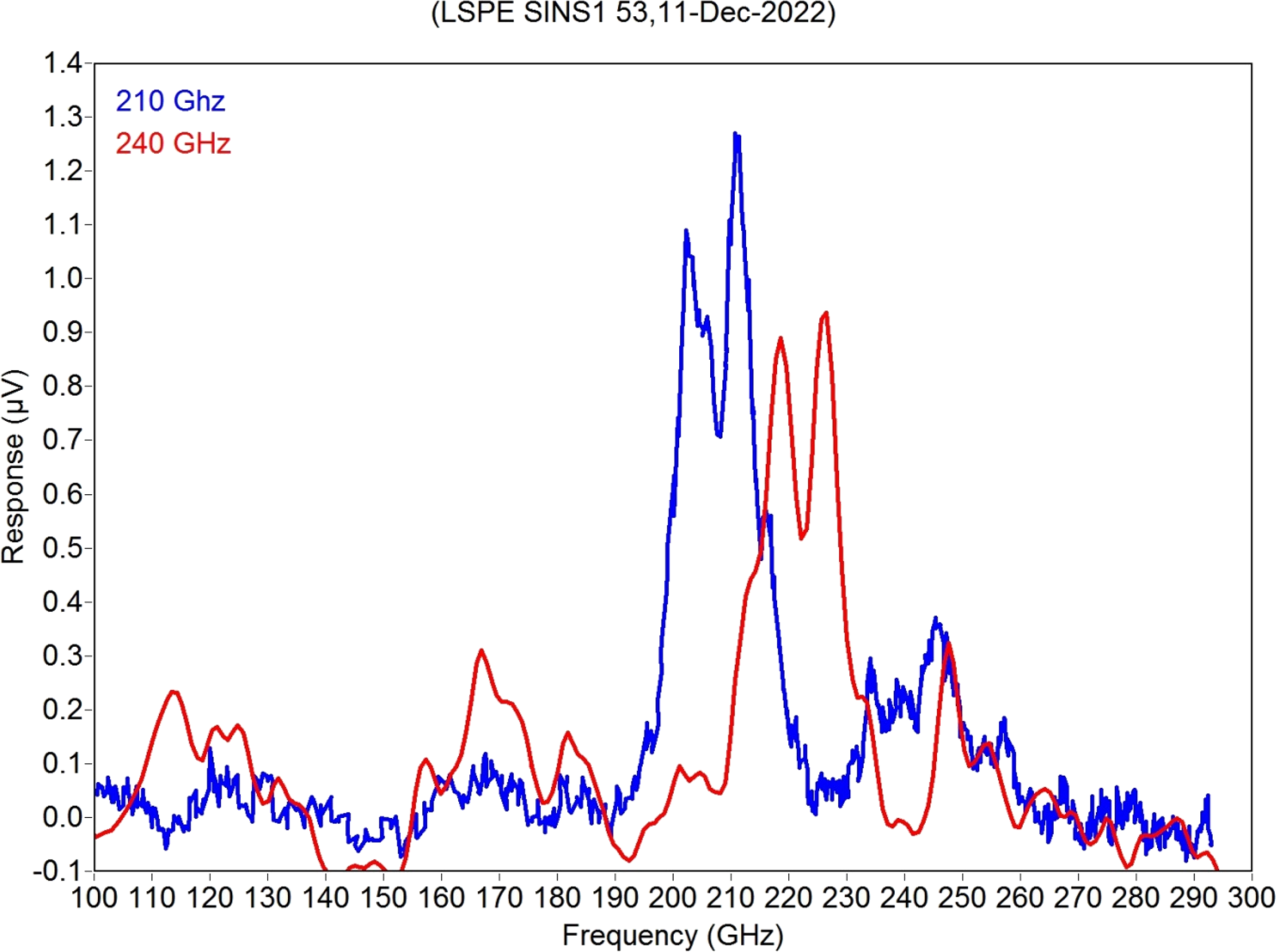
Measured frequency responses of two receiving structures of a receiving system prototype from the LSPE VB 210/240 SINS1 series.

**Figure 7:**
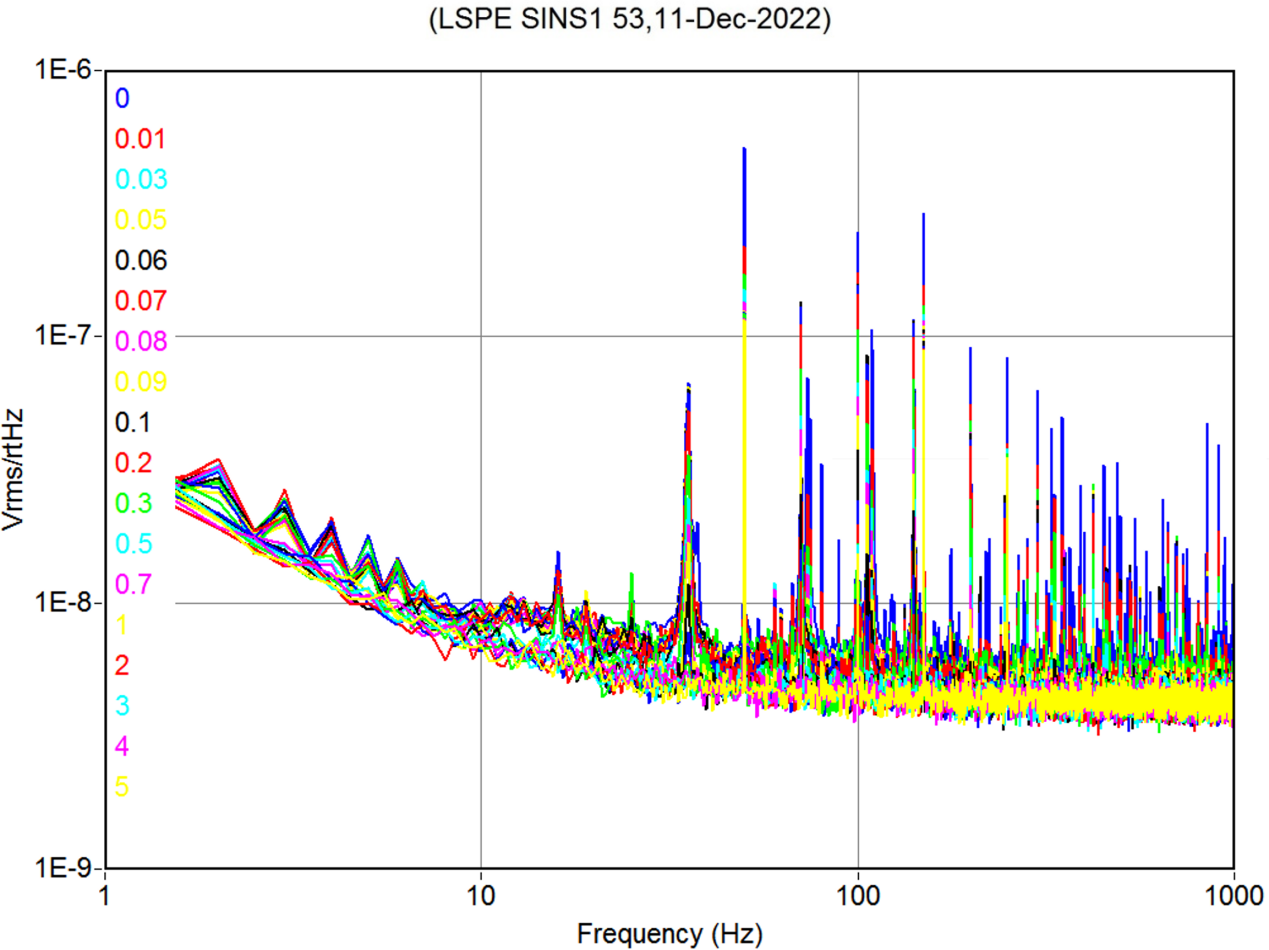
Noise spectra of a sample receiving system from the LSPE VB 210/240 SINS1 series 240 GHz array. To obtain the CEB bias current, featured in the *I*–*V* curve in Figure 5, from the voltages in the legend, one should divide the voltage by 3.75 × 10^6^.

Measurements of samples from the LSPE VB 210/240 SINS1 series, deposited at NNSTU, were performed in a sorption ^3^He cryostat at a temperature of 300 mK. Current–voltage (*I*–*V*) characteristics (Figure 5), frequency response (Figure 6), and noise at various bias currents (Figure 7) were measured. While the samples were designed to be used in the voltage bias mode with a SQUID readout system similar to the one described in [10], they were measured in the current bias mode with AD745 operational amplifiers due to the absence of proper equipment in our lab. Hence, the results might be different from the expected values.

As can be seen from Figure 5, the *I*–*V* characteristics of the 210 and 240 GHz arrays are quite close, which was not achieved at the first try. The normal structure resistance at 210 GHz (Figure 5, blue curve) was 200 Ω, the operating point resistance was 170 Ω, and the leakage resistance was 3.4 kΩ. The normal structure resistance at 240 GHz (Figure 5, red curve) was 310 Ω, the operating point resistance was 270 Ω, and the leakage resistance was 4.5 kΩ.

To study the resonant properties of the CEBs, we have used generators based on high-temperature YBCO Josephson junctions [27–29]. Long junctions based on YBCO thin films were fabricated by the preliminary topology mask method [30–31]. Specifically, YBCO film was deposited on a 24° [001]-tilt Zr_1−_*_x_*Y*_x_*O_2_ bicrystal substrate by magnetron sputtering with preliminary deposition of cerium dioxide CeO_2_ on the heated substrate as a buffer layer. The dimensions of the obtained Josephson junctions were 50 µm in length and 0.3 µm in thickness. At a temperature *T* ~ 2.7 K, the value of the critical current density was ~400 kA/cm^2^ with the *I*_C_*R*_N_ product of ~1.6 mV. The junctions were rather long since their lengths were much larger than the Josephson penetration depth λ_J_ < 1 µm (*T* = 2.7 K). The oscillator chip contains six JJ structures integrated into modified dipole antennas [29]. When the DC bias current is passed through the junction, the AC voltage arises, the frequency of which is strictly determined by the DC voltage in the junction through the Josephson relation. The electromagnetic wave produced in this way was effectively radiated by a dipole antenna at the edge of the JJ in the frequency range from 50 to 800 GHz [27]. As shown before [27–29], high-critical-temperature Josephson junctions are effective sources for studying the amplitude–frequency characteristics of various detector systems with broadband continuous frequency tuning in comparison with low-critical-temperature counterparts [32–33].

The experiment on the study of the amplitude–frequency characteristics of resonant bolometers was set up as follows: The generator chip with JJs was mounted on the sample holder with a 4 mm Si hyperhemispherical lens thermally coupled with a 2.7 K cryostat plate. The control magnetic field required to create a flux-flow regime in a long Josephson junction was created by a current flowing through the copper wire coil. A bolometer chip was placed on a 300 mK plate with a horn on the front side of the silicon substrate. The copper shield with a black body covered the receiver to avoid reflections.

Changing the central frequency of the generator radiation was performed by varying the bias current through the JJs and, thus, changing JJ voltage, such that *f*_YBCO_ = 2*eV*_YBCO_/*ћ*, where *e* is the elementary charge and *ћ* is Planck’s constant. The bolometer measurements were performed at constant current, that is, the change in voltage d*V*_CEB_ was measured as the response of the bolometer to an external gigahertz signal.

The generator antenna was investigated by measuring Shapiro steps [31] using a backward wave oscillator. In addition, the generator was independently calibrated by broadband CEB investigations in [27]. By substituting the Josephson relation between the frequency and voltage on the generator, we obtained the frequency response of the receiving resonant system.

On the frequency response plot, clearly pronounced peaks are visible in the region of 205 GHz (Figure 6, blue curve) and 225 GHz (Figure 6, red curve). A noticeable separation of frequency bands between arrays of antennas can be seen. We should note here that a certain frequency shift of the channels is due to improper Si substrate thickness (available in the clean room at that time), which was about 0.29 mm instead of the optimized 0.26 mm (see modelling results in Figure 2, dashed curves). In the next sample batch, the substrates must be properly etched to achieve the desired frequency range.

The noise level for a structure at 240 GHz at an operating point with a voltage of 400 µV at a frequency of 120 Hz is about 5 × 10^−9^ V/√Hz (Figure 7). These noise spectra were measured in the current bias mode with a readout scheme based on AD745 low-noise JFET operational amplifiers working at room temperature of 300 K. The estimated NEP for this measurement setup is about 5 × 10^−16^ W/√Hz with further improvement down to 1 × 10^−16^ W/√Hz in the voltage bias mode [24].

## Discussion

The results of measurements correspond to the simulation results rather well. There is a certain mismatch, which can be explained by the substrate thickness deviation from the desired value as well as improper barrier thickness due to variations in oxidation time, so the CEB SIN tunnel junction capacitance deviated from the calculated values. This latter deviation might directly affect the peak widths of measured frequency responses.

The other controversial part is the current bias measurement system, which is not optimal for such parallel arrays. Now we are working on a voltage bias scheme with low-noise readout. With the voltage bias mode, we can select the optimal operating point with minimal NEP, maximal responsivity, and minimal electron temperature simultaneously, while the current bias mode makes it complicated to find this optimum. Therefore, the NEP level should significantly improve with a voltage bias scheme. In case of the LSPE-SWIPE project power load, our estimations [24] demonstrate that, by aforementioned measurement system improvements, the photon NEP level, which is about 7 × 10^−17^ W/√Hz, can nearly be achieved.

## Conclusion

We have elaborated and tested the receiving arrays of dipole bow-tie antennas placed on a single silicon substrate to detect radiation at frequencies of 210 and 240 GHz. As a direct radiation detector, a novel concept of cold-electron bolometer with two SIN tunnel junctions and one SN contact is utilized. The amplitude–frequency characteristics measured with a YBCO Josephson junction oscillator show narrow peaks with band separation at 205 GHz for the 210 GHz array and at 225 GHz for the 240 GHz array. It is demonstrated that the undesired frequency shift is mainly due to improper Si substrate thickness in these test samples. The NEP level in the current bias mode is about 5 × 10^−16^ W/√Hz and can be further improved down to 1 × 10^−16^ W/√Hz in the voltage bias mode with low-noise SQUID readout.

## Data Availability

The data that supports the findings of this study is available from the corresponding author upon reasonable request.
